# Supplementary material for: Effect and Mechanism of Theaflavins on Fluoride Transport and Absorption in Caco-2 Cells
Source: Foods. 2023 Apr 1;12(7):1487. doi: 10.3390/foods12071487 (PMC10094491; doi:10.3390/foods12071487)
Supplement: Supplementary file 1 [file foods-12-01487-s001.zip › foods-2270438-supplementary.pdf]

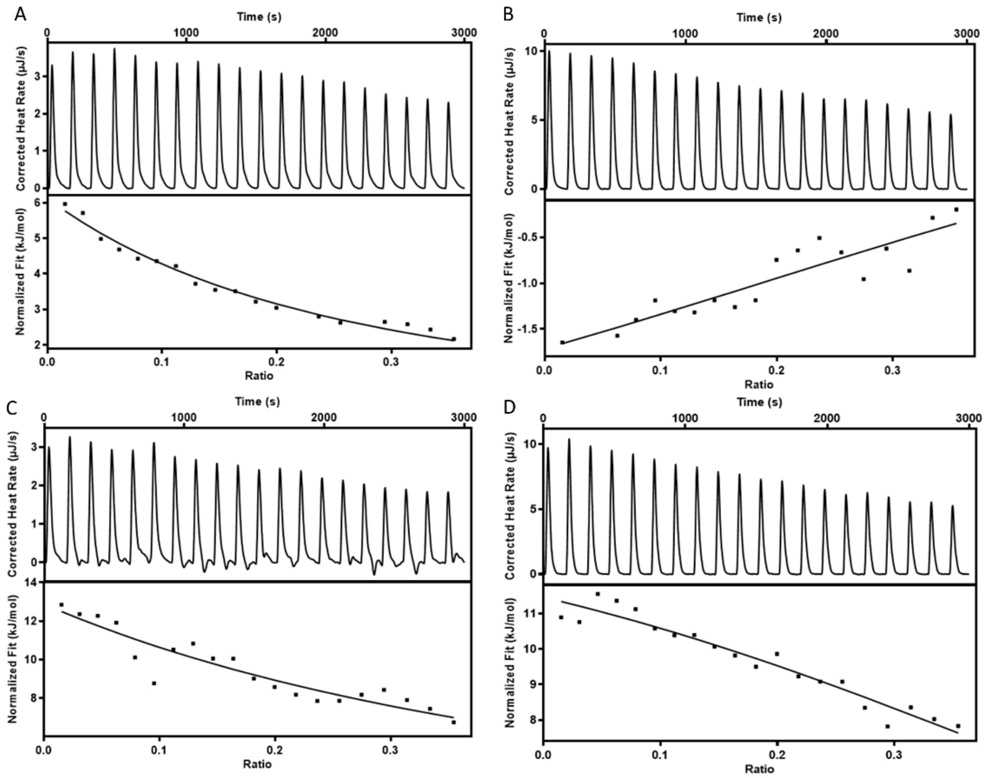

Figure S1. Thermal spectrum of F-+TFs titration process (up) and combined isotherm (down): (**A**) F-+TF, (**B**) F-+TF3G, (**C**) F-+TF3′G, (**D**) F-+TFDG.

Table S1. Primers used for qRT-PCR in this study

Primer name	Primer sequences (5'to 3')
ENSG00000123416-F	GTGCGTTACTTACCTCGACTCTT
ENSG00000123416-R	ACGCATAGTGGCTAGGGATTAG
ENSG00000184009-F	ATTGCCGACAGGATGCAGAA
ENSG00000184009-R	TGCTACGCATCTGCTGAGTC
ENSG00000085741-F	GCTCACCTGACTTCTGCATGAA
ENSG00000085741-R	GGGCCTCACTTGCAGACATAG

Table S2. TF3G distribution of the main peaks of Raman spectra with different concentrations of F- and their vibration mode

SERS band wavenumber (cm-1)	vibration mode
637	δ ring (monosubstituted benzene)
940	ring breathing
1074	υ ring (o-disubstituted benzene)
1263	ω ring (epoxy derivatives)
1540	ω (C=C coupling)
1640	ω (C=C)

υ: vibration; ω: wagging; δ: deformation.

Table S3. TF3'G distribution of the main peaks of Raman spectra with different concentrations of F- and their vibration mode

SERS band wavenumber (cm-1)	vibration mode
640	δ ring (monosubstituted benzene)
714	ω (C-C)
1265	ω ring (epoxy derivatives)
1309	δ (CH2)
1603	ω ring (twin peaks)
1717	ω (C=O)

υ: vibration; ω: wagging; δ: deformation.

Table S4. TFDG distribution of the main peaks of Raman spectra with different concentrations of F- and their vibration mode

SERS band wavenumber (cm-1)	vibration mode
775	υ ring (P-disubstituted benzene)
940	ring breathing
1259	ω ring (epoxy derivatives)
1527	ω ring
1642	ω (C=C)

υ: vibration; ω: wagging; δ: deformation.
